# Increasing trends of colistin resistance in patients at high-risk of carbapenem-resistant *Enterobacteriaceae*

**DOI:** 10.1080/07853890.2022.2129775

**Published:** 2022-11-14

**Authors:** Hadir A. El-Mahallawy, Marwa El Swify, Asmaa Abdul Hak, Mai M. Zafer

**Affiliations:** a Department of Clinical Pathology, National Cancer Institute, Cairo University, Cairo, Egypt; b Department of Microbiology and Immunology, Faculty of Pharmacy, Ahram Canadian University, Cairo, Egypt

**Keywords:** Carbapenem-resistant *Enterobacteriaceae*, aminoglycosides, tigecycline, colistin resistance, carbapenemase genes, healthcare-associated infections, cancer patients

## Abstract

**Introduction:**

Occurrence of colistin-resistant *Enterobacteriaceae* in response to the unregulated use of this antibiotic has been documented. This study reports an investigation of colistin resistance rates among carbapenem-resistant enterobacterial clinical isolates.

**Methods:**

A total of 196 multidrug-resistant Enterobacteriaceae isolates (*Klebsiella pneumoniae* (*n* = 100), *Escherichia coli* (*n* = 89) and *Enterobacter cloacae* (*n* = 7) were selected from Gram-negative isolates over one year. Susceptibility to antimicrobials was determined using Vitek2. Broth microdilution method was used to detect colistin antimicrobial susceptibility. Identification of ESBL and carbapenemases were both done phenotypically and by PCR.

**Results:**

All the studied isolates showed multidrug-resistant phenotypes with 51.5% resistance to carbapenems (meropenem, imipenem). Very low resistance rates towards tigecycline (*n* = 9) 4.6% were found. Thirty-nine isolates (19.9%) showed reduced susceptibility to colistin among the MDR isolates. Sixty-four isolates (32.7%) were ESBL producers. Hundred isolates (51%) were carbapenemase producers using Carba NP test. The PCR amplification results revealed that 40 isolates (20%) harboured NDM-1 and 40 isolates contained OXA-48-like gene. Coexistence of both (NDM-1 and OXA-48-like) was observed in nine (4.59%) isolates. A Statistically significant relationship was observed between carbapenem resistance and each of the followings; OXA-48 producers (*p*= .009), amikacin resistance (*p* = .000), gentamicin resistance (*p* = .032), tobramycin resistance (*p* = .000), and tigecycline resistance (*p*-value ≤ .001). A statistical significance was detected between ESBL-producing isolates and carbapenem susceptible isolates ESBL producers with *p* = 0.000.

**Conclusion:**

An alarming sign is the increasing colistin resistance rates among carbapenem-resistant isolates. Aminoglycosides are still a therapeutic option to decrease the use of colistin and avoid further development of resistance.

**KEY MESSAGES**
- High rates of colistin resistance among carbapenem-resistant *Enterobacteriaceae*.
- The choice of antibiotic is significantly associated with the clinical site of infection.
- Aminoglycosides are offered choices for treating multiple drug-resistant Enterobacteriaceae to preserve the colistin and carbapenems.

## Introduction

The unmonitored and indiscriminate use of antibiotics has resulted in contamination of environments, led to selective pressure on bacteria, and consequently increased the incidence of antibiotic resistance to every antibiotic introduced so far [1,2]. The rise in antibiotic resistance combined with the decline in the development of novel antibiotics is leading the world towards the catastrophic return of a pre-antibiotic era [3]. Carbapenem-resistant *Enterobacteriaceae* (CRE) has been classified as critical and requiring immediate attention by the WHO global priority list [4]. The expression of certain virulence determinants together with the dissemination of new clones makes treatment options challenging for multidrug-resistant *Enterobacteriaceae* infections [5]. CRE infections are often concomitant with high mortality, mainly due to delays in the administration of effective treatment. Carbapenem resistance in *Enterobacteriaceae* is mainly due to the expression of true carbapenemases [6]. On the other hand, carbapenem resistance due to acquired carbapenemases genes, are mostly plasmid-encoded, making them highly and rapidly transferable, at least within the enterobacterial species, and hence potentially responsible for outbreaks. They are also basically accompanied by multidrug resistance or pan-drug resistance [7]. One of the most clinically important carbapenemases is NDM-1 (New Delhi metallo-ß-lactamase) described in 2009 in *K. pneumoniae* and *E. coli* isolates from a patient in Sweden previously hospitalized in India [8]. Consequently, NDM producers in *Enterobacteriaceae* have been stated almost worldwide, including several countries in Asia, Africa, Australia, America, and Europe [9]. In 2003, the first recognized OXA-48 producer was a *K. pneumoniae* isolate recovered from Turkey [10]. OXA-48 producers have since been the source of healthcare-associated outbreaks and extensively reported in Turkey, then in North African countries and lately in the Middle East and India [11]. A recent online survey and literature review conducted in Egypt stated that NDM and OXA-48 genes were commonly reported in *Enterobacteriaceae* with an incidence of 26.04–68.88% and 30–58.62%, respectively [12]. The authors also reported that NDM genes were also commonly described in *E.coli* with a prevalence of 13.7–80.39% [12]. Additionally, a high prevalence of *bla_NDM_* and *bla_OXA-48_* genes was detected in *K.pneumoniae* at 20.9–100 and 0–80.65%, respectively [12]. The choice of the optimum antibiotic therapy to treat carbapenemase producers in healthcare-associated infections is primarily based on the antibiotic susceptibility testing results. In many situations, the antibiotic choice remains limited to colistin, parenteral fosfomycin, gentamicin, amikacin and tigecycline [13]. Therefore, the emergence of colistin resistance in carbapenem-resistant Enterobacteriaceae clinical isolates in the healthcare settings leaves the clinicians short of options in selecting an antibiotic regimen to treat these patients. The aim of this study was to investigate the incidence of colistin resistance in carbapenem-resistant enterobacterial isolates recovered from hospitalized patients in Cairo, Egypt. Further, the study aims to give an insight on the updates of the current situation for antimicrobial resistance in Egypt which shows a high endemicity of resistance.

## Methods

### Bacterial isolates

This was a retrospective cohort study including a total of 196 enterobacterial pathogens (*K. pneumoniae*, *E. coli* and *E. cloacae)* isolated from infectious samples of hospitalized cancer cases at National Cancer Institute, Cairo University, Egypt from January 2019 to December 2019. Other *enterobacteriaceae* members e.g. *Salmonella*, *Shigella*, *Serratia* etc. are rarely detected in our cases, so were excluded from the study. This study was approved in January 2019 by the medical ethics committee of the National Cancer Institute, Cairo University, Egypt. Identification of the collected isolates was examined by biochemical tests using standard microbiological procedures [14]. Further bacterial identification was performed using Vitek2 Compact (bioMerieux, Marcy l’Étoile, France) following the instruction of the manufacturer.

### Phenotypic evaluation of antibiotic resistance

The antibiotic resistance profile of all the collected isolates were determined by Vitek2 (bioMerieux, Marcy l’Étoile, France). These tests were performed following the Clinical and Laboratory Standards Institute (CLSI) guidelines [15]. Susceptibility was interpreted according to the criteria of the CLSI 2020 [15], except for susceptibility to tigecycline, which was interpreted following the FDA criteria (susceptible: ≤2 mg/L; resistant: ≥8 mg/L) for Enterobacteriaceae [16]. *Escherichia coli* strain ATCC25922 was used as reference control strain.

### Colistin broth microdilution testing

The minimum inhibitory concentrations (MIC) of colistin were determined using the microbroth dilution method according to EUCAST recommendation testing of *Enterobacteriaceae* [17]. Un-supplemented cation adjusted Mueller-Hinton broth (CAMHB) is used. The test was performed using plain 96-well polystyrene microwell-plates containing two-fold dilutions of colistin sulphate salt (0.12–64 µg/ml) (Sigma-Aldrich, St. Louis, MO, USA) in CAMHB. *Escherichia coli* ATCC 25922 was used as quality control strain.

### Phenotypic detection of ESBL and carbapenemase production

The phenotypic detection of ESBL production was tested using double-disc synergy test.

The biochemical Carba NP test was performed following the CLSI guidelines with slight modifications for screening carbapenemase production [15].

### Molecular screening of carbapenemase genes

Characterization for carbapenemase encoding genes *NDM, VIM, IMP, SIM, GIM, SPM, OXA-48, and KPC* by two multiplex polymerase chain reactions (PCR) as previously mentioned was conducted on all the isolates [18,19].

### Statistical analysis

Statistical Package for Social Science (SPSS®) Statistics version 22 (IBM-SPSS Inc., Chicago, IL, USA) was used for data analyses. Frequency and percentage were used to state qualitative data. Relations between qualitative variables were assessed using the Pearson’s chi-square test and the Fisher’s exact test. A value of *p* < 0.05 were considered statistically significant.

## Results

**Figure 1.**
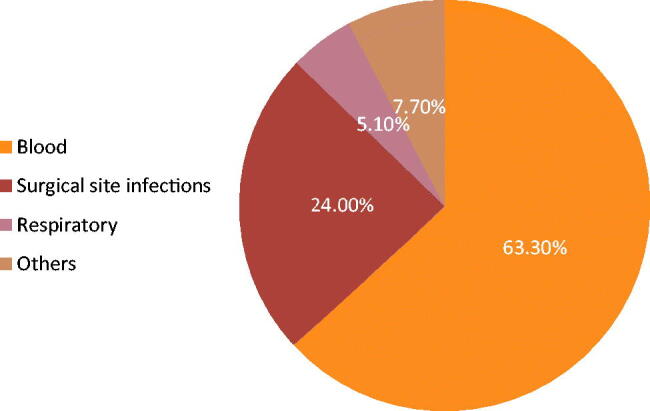
Source of specimen.

This study was conducted in the microbiology laboratory at the National Cancer Institute (NCI), Cairo University, between January and December 2019. NCI is a tertiary referral hospital receiving cancer patients from all over Egypt. In total, 196 multidrug-resistant enterobacterial isolates were collected during the study period. These were recovered from 196 different hospitalized cancer adult patients with either haematology malignancy or solid tumours with age ranging between 18 and 55 years old. Of these, 55.6% (*n* = 109) were males and 44.4% (*n* = 87) were females. The collected isolates included 100 (51%) *K. pneumoniae*, 89 (45.4%) *E. coli* and seven (3.6%) *E. cloacae*. The recovered infectious isolates were obtained from different clinical sources. Most of the isolates were recovered from blood cultures 62.6% (*n* = 124), surgical site infections specimens (pus, wound) 24% (*n* = 47), sputum and chest tube 5.1% (*n* = 10), and specimens from other sites 7.7% (*n* = 15) (Figure 1). The chest tubes were inserted in cases of clinically suspected lower respiratory tract infections, i.e. they had infections prior to chest tube insertion. Besides, the sample was obtained in the first two to three days of insertion and the organism isolated was a known pathogen with detected antibiotic resistance.

### Phenotypic detection of antimicrobial resistance, ESBLs and carbapenemase production

**Figure 2.**
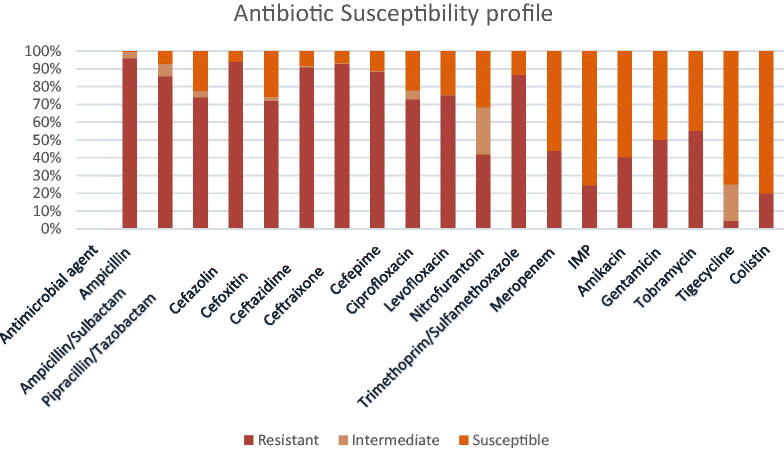
Antimicrobial resistance rates of MDR *Enterobacteriaceae.*

Results of susceptibility testing using VITEK-2 automated system revealed that all the isolates were multidrug-resistant (resistance to at least one agent in three or more antimicrobial categories) [20]; among the antimicrobial agents tested, high resistance rates were observed for ampicillin (96%), cefazolin (94%), ceftriaxone (93%), ceftazidime (91%), cefepime (88%), trimethoprim/sulfamethoxazole (87%), ampicillin/sulbactam (86%), levofloxacin (75%), piperacillin/Tazobactam (74%), ciprofloxacin (73%), followed by tobramycin (55%) and gentamicin (50%). Low resistance rates to meropenem (44%), nitrofurantoin (42%), amikacin (40%), imipenem (24%), and tigecycline (4.6%) were found (Figure 2). No significant difference was found between antibiotic resistance profile and carbapenemase genes (NDM and OXA-48).

**Figure 3.**
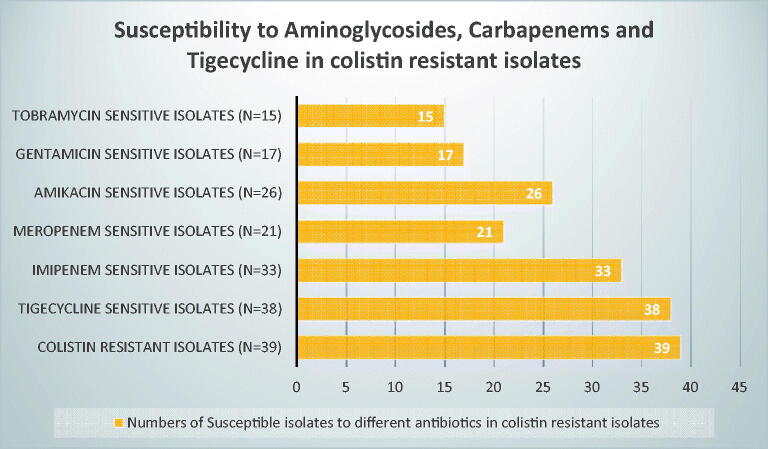
Number of isolates showing susceptibility to other antibiotics while being resistant to colistin.

It is worth mentioning that colistin-resistant isolates retained susceptibility to at least one of these antibiotics (tigecycline, carbapenems and aminoglycosides); out of the 39 colistin-resistant isolates, 38, 33, 26, 21, 17, 15 isolates were susceptible to tigecycline, imipenem, amikacin, meropenem, gentamicin, and tobramycin respectively (Figure 3).

Phenotypic detection for ESBL production was done using double-disc synergy test and showed that 64 isolates (32.7%) were ESBL producers whereas 132 isolates were negative for ESBL production.

The results of the Carba NP test for Enterobacteriaceae isolates showed that 100/196 (51%) isolates were carbapenemase producers, of which 94 isolates are carbapenem resistant, whereas 96/196 (49%) isolates were carbapenemase non-producers.

The results of broth microdilution showed that 21 *K.pneumoniae* isolates and eighteen *E.coli* were colistin-resistant (19.9%) with MIC range of range of >2–32 µg/ml.

### Genotypic detection of carbapenemase genes

**Figure 4.**
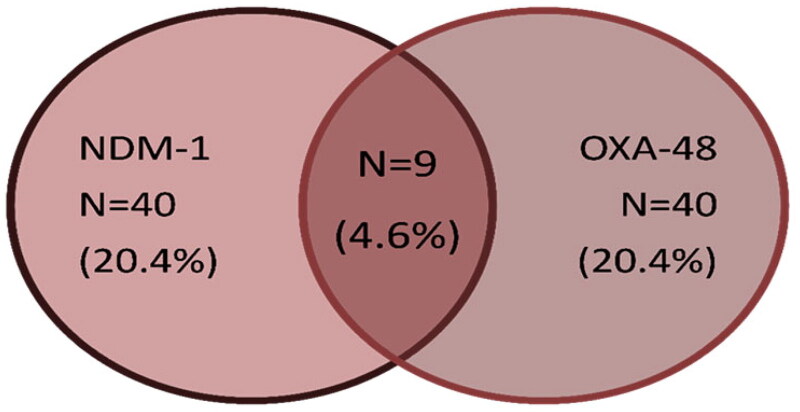
Occurrence of carbapenemases genes among MDR *Enterobacteriaceae.*

A multiplex PCR was performed to detect the most common carbapenemase and metallo-beta lactamase genes, the results showed that 71/196 (36.2%) isolates harboured carbapenemase genes; 40/196 (20.4%) showed amplification of the 238 bp fragment for *bla_OXA-48_* and 40/196 (20.4%) showed amplification of the 521-bp fragment for *bla_NDM-1_*. Double existence of both *bla_OXA-48_* and *bla_NDM-1_* were detected in only nine isolates (Figure 4). None of the other carbapenemase or metallobeta-lactamase genes (KPC, VIM, IMP, SIM, GIM, SPM) were identified in the studied enterobacterial isolates. Notably, a statistical significance of imipenem resistance and NDM producers was detected (*p* = .032). Additionally, a significant relation was also found between meropenem resistance, carbapenem resistance and OXA-48 production with p-value 0.021and 0.009 respectively (Table 1).

**Table 1.** Relation between carbapenem resistance and associated characteristics.

Associated characteristic			Carbapenem sensitive *N* (%)	Carbapenem resistant N (%)	*p*-Value
Organism					
* E. coli (n* = 89)			49 (55%)	40 (45%)	
* K.pneumoniae* (*n* = 100)			42 (42%)	58 (58%)	0.185
* E. cloacae* (*n* = 7)			4 (57%)	3 (43%)	
Gender					
Male (*n* = 109)			51 (46.8%)	58 (53.2%)	0.598
Female (*n* = 87)			44 (50.6%)	43 (49.4%)	
Source of specimen					
Blood (*n* = 124)			50 (40.3%)	74 (60%)	
Pus, wound (*n* = 47)			31 (66%)	16 (34%)	**0.003**
Sputum, chest (*n* = 10)			8 (80%)	2 (20%)	
Others (*n* = 15)			6 (40%)	9 (60%)	
ESBL					
Positive (*n* = 46)			56 (88%)	8 (12%)	**0.000**
Negative (*n* = 132)			39 (30%)	93 (71%)	
Antibiotics					
Aminoglycosides					
Amikacin		S (*n* = 117)	89 (93.7%)	28 (27.7%)	**˂0.001**
		R (*n* = 79)	6 (6.3%)	73 (72.3%)	
Gentamicin		S (*n* = 98)	55 (57.9%)	43 (42.6%)	**0.032**
		R (*n* = 98)	40 (41%)	58 (59%)	
Tobramycin		S (*n* = 88)	62 (65.3%)	26 (25.7%)	**˂0.001**
		R (*n* = 108)	33 (34.7%)	75 (74.3%)	
Tigecycline		S (*n* = 147)	85 (89.5%)	62 (61.4%)	**˂0.001**
		R (*n* = 49)	10 (10.5%)	39 (38.6%)	
Colistin		S (*n* = 157)	73 (76.8%)	84 (83.2%)	0.268
		R (*n* = 39)	22 (23.2%)	17 (16.8%)	
Carbapenemase genes					
NDM		Positive (*n* = 40)	18 (18.9%)	22 (21.8%)	0.623
		Negative (*n* = 156)	77 (81.1%)	79 (78.2%)	
OXA-48		Positive (*n* = 40)	12 (30%)	28 (70%)	**0.009**
		Negative (*n* = 156)	83 (53%)	73 (47%)	

Isolates with intermediate susceptibility towards tigecycline were considered resistant.

Bold values indicate statistically significant results.

### Carbapenem resistance and associated characteristics

**Figure 5.**
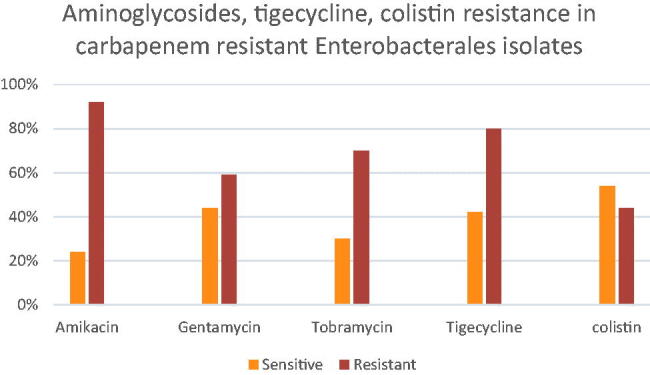
Aminoglycosides, tigecycline and colistin resistance in carbapenem-resistant *Enterobacteriaceae* (40 [20.4%] showing intermediate resistance to tigecycline were considered resistant).

No significant difference between carbapenem resistance and type of organism (*E. coli*, *K.pneumoniae* and *E.cloacae*) was found (*p* = .185). Similarly, no significant difference between carbapenem resistance and gender (*p* = .598). Carbapenem resistance rates were significantly higher in samples received from blood (*p* = .003). ESBL production was significantly observed in carbapenem-sensitive isolates (*p* = .000) (Table 1). Figure 5 shows the relation between carbapenem resistance and resistance to different antibiotics. Significant relation of carbapenem resistance and amikacin resistance (*p* < .001) was observed, carbapenem resistance and gentamicin resistance (*p* = .032) were significantly related. A similar significant relation between carbapenem resistance and tobramycin resistance (*p* < .001) was found. Tigecycline resistance and carbapenem resistance was significantly associated (*p* < .001) (Figure 5). No relation was observed between carbapenem resistance and colistin-reduced susceptibility (*p* = .268) (Table 1).

Table 2 clearly demonstrates the difference in antibiotic choice according to the clinical site of infection, certain antibiotics can be more effective in different sites of infections. For example,74% of surgical site infections are susceptible to amikacin, whereas 53% of blood samples are amikacin susceptible (Table 2).

**Table 2.** Antibiotic patterns according to clinical sites of infection.

Source of specimen	ESBL producing isolates (*n* = 64)	Carbapenemase producing isolates (*n* = 100)	Antibiotic susceptibility patterns					
			Amikacin	Gentamicin	Tobramycin	Tigecycline	Colistin	Carbapenem
			Susceptible isolates	Susceptible isolates	Susceptible isolates	Susceptible isolates	Susceptible isolates	Susceptible isolates
			(*n* = 117)	(*n* = 98)	(*n* = 88)	(*n* = 147)	(*n* = 157)	(*n* = 95)
Blood (*n* = 124)	31 (25%)	72 (58%)	66 (53%)	64 (52%)	45 (36%)	87 (70%)	103 (84%)	50 (40%)
SSIs (*n* = 47)	24 (51%)	16 (34%)	35 (74%)	22 (49%)	29 (62%)	43 (91%)	36 (77%)	31 (66%)
Sputum and Chest (*n* = 10)	5 (50%)	2 (20%)	9 (90%)	6 (60%)	9 (90%)	7 (70%)	8 (80%)	8 (80%)
Other sites (*n* = 15)	4 (27%)	10 (67%)	7 (47%)	6 (40%)	5 (33%)	10 (67%)	10 (67%)	6 (40%)

Isolates with intermediate susceptibility towards tigecycline were considered resistant.

## Discussion

Understanding the local epidemiology, resistance mechanisms among different antimicrobials available, and type of carbapenemase genes in carbapenem resistant-Enterobacteriaceae are mandatory to set strategies of therapy in critically ill patients. Limited antibiotic options for the treatment of MDR and/or carbapenem-resistant Enterobacteriaceae are currently available. Herein, we determined the incidence of colistin resistance rates, antimicrobial resistance characteristics, and carbapenemase types in MDR Enterobacteriaceae isolates. Cancer patients are uniquely threatened by multidrug-resistant Gram-negative bacteria because they are subjected to multiple antibiotic courses coinciding with repeated chemotherapy sessions [21]. In this study, MDR Enterobacteriaceae were isolated with high frequency from clinical samples of 196 hospitalized cancer patients with haematologic malignancies and solid tumours during the study period. Consistent with other reports, our study reflects that *K.pneumoniae* accounts for the largest proportion of MDR Enterobacteriaceae [12]. Multidrug-resistant Gram-negative pathogens are an important cause of bloodstream infections causing sepsis in cancer patients. In this study, a major percentage of the isolates were obtained from blood specimens (63.6%), followed by isolates recovered from surgical site infections (24%) suggesting that multidrug-resistant Enterobacteriaceae bloodstream infections is a major problem in cancer patients. This result agrees with earlier reports stating that blood specimens were the most dominant among carbapenem-resistant Enterobacteriaceae [22,23]. Antimicrobial susceptibility testing indicated that the studied Enterobacterales strains exhibited high resistance to a variety of tested antibiotic classes. In this study, a remarkably high level of colistin resistance was detected among the studied MDR Enterobacteriaceae isolates with a prevalence of 19.9%. An increased rate of colistin resistance compared to our previous study conducted in the same hospital (colistin resistance: 8.8%) in 2019 might be due to the widespread use of colistin in our hospital in high-risk patients due to lack of other treatment options [24]. Moreover, a much lower rate of colistin resistance was reported by an earlier Egyptian study in 2016 (4.4%) [25]. Additionally, unpublished data on the use of colistin in our hospital (National Cancer Institute) showed that colistin was added to 56 febrile neutropenic cancer patients out of 162 patients that were infected with MDR Enterobacteriaceae i.e. 35% of infectious episodes necessitated the addition of colistin. Fortunately, in this study all the colistin-resistant isolates retained susceptibility to other classes of antibiotics. However, this is not the situation in countries that banned the use of colistin alongside the emergence of plasmid-encoded mcr genes. Reports of significant reduction of colistin resistance rates from such countries have been described [26–28]. In addition, countries that have never accepted the handling of colistin in animal production have repeatedly stated lower colistin-resistant rates [26]. Alarming high rates of carbapenem resistance detected among the collected MDR isolates in which 58 *K.pneumoniae*, 40 *E.coli* and 3 *E.cloacae* isolates were carbapenem resistant with total 101 carbapenem-resistant isolates (51.1%). In this study, 16.8% enterobacterial isolates showed resistance to both colistin and carbapenems (Table 1). This further limit treatment options. It is noteworthy to mention that carbapenemases represent the most important mechanism of resistance in Enterobacterales, since they are highly transferable therefore are considered to exert more threat on public health than noncarbapenemase producers and they are associated with multi-drug resistance [8].Consistent with these previous reports, our results showed that 40 isolates (22 *K.pneumoniae*, 16 *E.coli* and 2 *E. cloacae*) contained OXA-48; 28 of which were carbapenem resistant. Forty isolates (20 *K.pneumoniae*, 18 *E.coli* and 2 *E. cloacae*) as well harboured NDM, 22 isolates showed reduced susceptibility towards carbapenems. The association of OXA-48 and NDM-producing isolates was observed in 9 isolates (4.6%) (Figure 3). The remaining carbapenem-resistant isolates that were negative for carbapenemase genes production might be due to existence of novel carbapenemase genes or may be due to existence of other resistance mechanism as porin deficiency which is non-transferable. Alarmingly, OXA-48 and NDM were detected in Carbapenem susceptible isolates; 12 OXA-48 producing isolates were susceptible to carbapenems and 18 NDM-positive isolates showed susceptibility towards carbapenem as well. Moreover, the results of the Carba NP test for Enterobacterales isolates in this study showed that 100/196 (51%) isolates were carbapenemase producers, of which 94 isolates are carbapenem resistant, whereas 96/196 (49%) isolates were carbapenemase non-producers. These findings indicate the silent dissemination of carbapenemase genes in our hospital setting because the antibiotic susceptibility result by disc diffusion and/or MICs remain low and the phenotypic detection for carbapenemase production may be negative as well, therefore, it is advisable to screen all MDR isolates whether carbapenem susceptible or resistant for carbapenemase gene production using PCR based methods in order to minimize their silent spread in the hospital settings which is clearly addressed in this study. This study reported 64 (23.4%) isolates as extended-spectrum beta-lactamase (ESBL) producers. *E. coli* was the most common ESBL active (*n* = 34) followed by *K.pneumoniae* (*n* = 29) and one *E.cloacae* was ESBL producer. Results of several reports are in accordance with our results on ESBL activity, where they stated that ESBL activity was more frequent in *E. coli* [29,30].

In this study, ESBL production was associated with eight carbapenem-resistant isolates, this finding represents a major threat as carbapenem is supposed to be the drug of choice in treatment of ESBL-producing Enterobacteriaceae. However, the majority of ESBL-producing Enterobacterales detected in this study were carbapenem susceptible (*n* = 56, 88%) and susceptible to amikacin as well (*n* = 59, 92.2%). This result clearly explains that EBSL-producing Enterobacterales usually stay susceptible to carbapenems [31]; and further highlights the current treatment options available in treatment of ESBL-producing Enterobacteriaceae in our hospital. Though, the treatment options for carbapenem-resistant Enterobacterales infections is challenging, this study investigated the antimicrobial activity of tigecycline, colistin, aminoglycosides against carbapenem-resistant Enterobacterales strains. The highest activity against carbapenem-resistant Enterobacterales demonstrated by colistin (83.2%), followed by tigecycline which exhibited 61.4% sensitivity against carbapenem-resistant Enterobacterales. Then aminoglycosides revealing the highest activity being displayed by gentamicin 43.9%, then tobramycin and amikacin with sensitivity 29.5% and 23.9% respectively. A high rate of resistance towards amikacin could be explained by the increased use of amikacin as combined therapy with colistin in our hospital.

Currently, few of the new antibiotic combinations promising to be effective against CRE are available in Egypt, yet their very high price is limiting their use. In general, until more definitive evidence is declared, optimizing the use of novel and old antibiotic options is vital [32].

Limitations of the current study, mechanisms of colistin resistance were not investigated, genotyping to detect the clonal relationship of the isolates were not studied here, as the main purpose was to investigate the current situation of the available antibiotics for treating the problematic carbapenem-resistant Enterobacterales.

## Conclusion

The increase in the incidence of MDR Gram-negative pathogens in critically ill patients is worrying; complicating the available treatment options. The knowledge of local epidemiology of carbapenem-resistant Enterobacterales is crucial in order to pick the best treatment decisions, therefore, minimize the spread of these problematic pathogens in the hospital settings.

## Data Availability

All data generated or analysed during this study are included in this published article.
